# Effectiveness of Earplugs and Eye Masks on Sleep Quality and Fatigue Among Nonventilated Patients in an Intensive Care Unit

**DOI:** 10.7759/cureus.63628

**Published:** 2024-07-01

**Authors:** Seetha Lakshmi Avudaiappan, Sivagami Govindaraj, Geetha Poomalai, Sumathi Mani

**Affiliations:** 1 Department of Nursing Foundation, Faculty of Nursing, Sri Ramachandra Institute of Higher Education and Research, Chennai, IND; 2 Faculty of Nursing, Mount Zion College of Nursing, Pilivalam, IND

**Keywords:** hemodynamic parameters, sleep quality, intensive care unit, eye mask, earplugs

## Abstract

Background: Sleep is a basic physiological need and is imperative for healing and rejuvenation. However, the environment of the intensive care unit (ICU), including loud sounds and bright lights, can undermine patients' sleep quality.

Aim: This study was designed to determine the effectiveness of overnight use of earplugs and eye masks to improve sleep quality and potentially influence hemodynamic parameters and mitigate fatigue among nonventilated patients in an ICU.

Materials and methods: This experimental study was conducted among 84 nonventilated patients in an ICU. The patients were evenly divided between an intervention group (n=42) and a control group (n=42). The intervention group patients received earplugs and eye masks for three consecutive nights from 10:30 pm to 6:30 am, while the control group patients received routine care. Clinical data, responses to the Richards-Campbell sleep questionnaire, and rankings on a numerical fatigue scale were collected before (pretest) and after the intervention each night and the next morning (posttests 1, 2, and 3).

Results: The results revealed a significant reduction in fatigue. At pretest, fatigue scores in the study group were 4.19±1.64. The score was significantly reduced to 3.40±1.39 at posttest 1 and then to 2.21±1.00 at posttest 3 (p<0.0001). The sleep quality for the study group showed a significant improvement from the pretest score of 43.73±8.27 to 60.35±6.85 at posttest 3 (p<0.001), whereas the control group had slightly worse sleep quality, with 40.64±8.67 at pretest and 45.63±6.95 at posttest 3.

Conclusions: Continuous patient monitoring is an essential nursing care activity in ICUs while ensuring good-quality sleep promotes healing and reduces fatigue. Sleep quality can be supported by devices such as earplugs and eye masks to limit undue disturbances in the ICU settings.

## Introduction

Hospitalization can have an adverse impact on sleep, particularly after admission to the intensive care unit (ICU), because monitoring and emergency management of a condition can make sleep difficult. A lack of sleep among ICU patients can affect respiratory muscles and pulmonary reserves, elevate blood pressure, and alter metabolic functions and neurocognitive responses [1]. A comparison of the sleep experience in terms of quantity and quality revealed that the in-hospital sleep quality was poor compared to the sleep quality at home. Factors contributing to poor sleep were disturbances for care, noise from other patients, and the hospital environment. Qualitative inputs for improving sleep included reducing disruptive noise from devices such as alarms, doors, trolleys, other patients, and staff [2]. An observational study in ICUs identified that the mean noise levels were high during the day and at night, compared with World Health Organization (WHO) guidelines of 35 dB for daytime and 30 dB at night [3], and the noise levels had adverse impacts on anxiety and sleep quality [4]. Similar findings were reported for a burn ICU, where the daytime and nighttime noise levels were high. Proposed solutions to limit the disruption to patients and facilitate a healing ICU environment included providing earplugs to patients to mute their perception of sounds or using alternative measures for alarms such as light or vibrations [5].

Multiple studies have assessed sleep and the factors affecting it in ICUs. Sleep and daytime sleepiness in the ICU can be influenced by environmental factors, such as noise, light, nursing care, diagnostics, measurement of vital signs, blood sample collection, and administration of medication, as well as nonenvironmental factors, such as the use of sleep medications, health conditions and their severity, and the use of medications such as steroids and benzodiazepines that affect sleep quality [6]. Noise and light were found to have a significant impact on sleep, followed by factors such as nursing interventions, medication administration, and checking vital signs [7]. In addition, low light levels during both the day and night and brief exposures to bright light during the night could affect the circadian rhythm of ICU patients [8].

The barriers identified for sleep disruptions in the ICU were discomfort, pain, use of medical devices such as a mechanical ventilator, and medications [9] and stress or anxiety [10]. In addition, sleep quality was found to be poor among patients with chronic illness APACHE II and simple acute physiology score 3, those with older age, and those on mechanical ventilation [11]. Lê Dinh et al. [12] found that the total sleep time among ICU patients was 4-5 hours, with poor quality of sleep seen in patients with higher SOFA scores, anxiety, dyspnea at the time of admission, and air leaks and noninvasive ventilation failure. Hospitalized surgical patients were found to have poor sleep quality and experienced more fatigue than patients admitted to internal clinics [13].

A meta-analysis of 20 studies identified factors such as alarms, sound and patient care activities, nighttime care, and physical and physiological alterations. Exposure to noise and light led to poorer perceived sleep quality and sleep disruption among ICU patients [14].

Strategies to facilitate sleep have been suggested by ICU patients and staff [15]. The patient's suggestions for improving sleep include closing doors or using blinds to avoid unnecessary interruptions, the use of sleep medications, and the reduction of lights. Staff suggestions were to lower the volume of the alarms, to remedy the reason for sleep disruption quickly, and to offer earplugs to the patients. Johansson et al. [16] also suggested that improving staff knowledge about noise in the ICU could foster clinical improvement.

The primary aim of the present study was to determine the effectiveness of using earplugs and eye masks on sleep quality and to assess the influence on hemodynamic parameters and fatigue among nonventilated ICU patients.

## Materials and methods

An experimental pretest and posttest control group design was selected for the study. The sample size was calculated based on a previous study [17], based on a 5% level of significance and power of 80%. The researcher selected the sample after 24 hours of admission to the ICU on each day and allocated the patients available on that day to the study and control groups. This was repeated for the entire duration of data collection. The total sample size was 84 patients, evenly divided between a treatment group (n=42) and a control group (n=42). Data were collected from October 17 to November 27, 2022. The study was conducted in a medical ICU with 16 beds and a critical care ICU with 16 beds.

Patients were evaluated for inclusion based on the following criteria: >21 years of age, no sedative medications or opioids for the past 24 hours, not mechanically ventilated, not experiencing any auditory/visual problems, and Glasgow coma scale >13. Patients diagnosed with encephalopathy, severe dementia, encephalitis, increased intracranial pressure, or severe hemodynamic instability; those with a previously diagnosed sleep disorder; and those on sleep medication were excluded from the study. Participants were randomly assigned to either the study group or the control group based on drawing odd or even numbers. 

The intervention included the use of earplugs and eye masks. The earplugs were rated for reducing the noise level to less than 30 dB, which is the WHO standard for ICUs [4]. The eye mask was constructed from a double layer of black cotton fabric and had adjustable straps to provide a close fit to patients' faces. The earplugs and eye masks were pilot-tested and found to be appropriate and comfortable. The purpose of the study, their option to participate in the study, and their right to withdraw from it at any time were explained to the patients in their own spoken language. The intervention was explained to the patients, and written consent was obtained from all study participants. The ICU patients in the study group were given earplugs and eye masks after their first 24 hours of admission and asked to wear them from 10:30 pm to 6:30 am. Patients could remove their eye masks and earplugs if they had any discomfort or needed something from the staff. This information was repeated for day 1, day 2, and day 3 for the same group of patients.

Demographic data were collected from all patients and included age, sex, place of residence, educational status, family income, type of family, social support, marital status, occupation, and socioeconomic status. Data were also collected for clinical variables, including body mass index, sleep disturbance factors, alcohol use, current smoking status, diet pattern, history of previous illness, duration of current illness, and previous ICU experience. Hemodynamic parameters were measured on all three days and included blood pressure, heart rate, respiration, and SPO2.

Fatigue was assessed by using a numerical fatigue scale [18] based on 0- to 10-point ratings of the level of fatigue, with higher scores indicating greater fatigue. The subjective sleep quality was determined using the Richards-Campbell sleep questionnaire which has six items and evaluates nighttime sleep on a visual analog scale (VAS) of 100 for best sleep and 0 for worst sleep. Each component such as sleep depth had scores ranging from deep sleep (100) to light sleep (0); latency (time to fall asleep) ranging from fell asleep immediately to never could fall asleep; number of awakenings ranging from awake very little to awake all night long; efficiency (percent of time awake) ranging from got back to sleep immediately to couldn't get back to sleep; quality ranging from a good night's sleep to a bad night's sleep; and perceived nighttime noise ranging from very quiet to very noisy along the continuum of 100 to 0. The scores out of 100 are then divided by five to obtain the total score, and a lower score indicates poor quality of sleep and a higher score indicates good quality of sleep.

Data were analyzed using IBM SPSS Statistics for Windows, Version 23.0 (Released 2015; IBM Corp., Armonk, New York, United States). The paired t-test was used to compare changes within groups over three posttests, and the independent t-test was used to identify differences between the study and control groups. Repeated measures ANOVA was used to determine the effect of earplugs and eye masks on sleep quality, hemodynamic parameters, and fatigue over three posttests.

## Results

The mean age of the participants was 64 years (range 36-80 years) in both groups (p=0.767). Around 38.09% in the study group and 28.63% in the control group were between 66 and 75 years of age. Twenty-six (61.9%) patients in the study group and 29 (69.0%) in the control group were male, and 25 (59.5%) patients in the study group and 30 (71.5%) in the control group resided in an urban area. In addition, 24 (57.1%) patients in each group were supported by their children as the primary caregiver. Thirty (71.42%) and 24 (57.14%) of the patients had comorbidities such as type 2 diabetes mellitus, hypertension, or both in the study and control groups, respectively. Around 30% and 41.66% of the study and control groups had a duration of illness between one and five years as depicted in Table 1. The reason for admission in both groups included diabetes, hypertension, acute and chronic kidney disease, anemia, meningitis, cellulitis, diabetic ketoacidosis, upper GI bleeding, chronic liver disease, previous cerebrovascular accident with hypertension, chronic obstructive pulmonary disease, and chronic liver disease.

**Table 1:** Distribution of demographic variables of the ICU patients (n=84 (42+42)) ***p<0.001; **p<0.01 ICU: intensive care unit

Demographic variables	Study group		Control group		P-value
	f	%	f	%	
Age (in years)					
36-45	2	4.4	3	7.14	0.767
46-55	6	14.28	10	23.8	
56-65	9	21.43	8	19	
66-75	16	38.09	12	28.63	
>75	9	21.44	9	21.43	
Gender					
Male	26	61.9	29	69.0	0.491
Female	16	38.1	13	31.0	
Residence					
Rural	17	40.4	12	28.57	
Urban	25	59.5	30	71.5	0.251
Comorbidities					
Diabetes mellitus/hypertension/both	30	71.42	24	57.14	
None	12	28.57	18	42.85	0.63
Duration of illness					
Less than 1 year	8	26.67	7	29.16	
>1-5 years	9	30.00	10	41.66	0.839
6-10 years	11	36.67	7	29.16	
More than 10 years	2	6.66	0	0	

The median ICU length of stay was two days (range 1-7 days; p=0.004). In both groups, 23 (54.8%) patients had no ICU experience. The demographic and clinical variables were homogenous for the study and the control groups. All 42 patients in the study group completed the Richards-Campbell sleep questionnaire survey questionnaire, and none of them reported any discomfort in using the earplugs or eye mask.

Hemodynamic monitoring

As shown in Table 2, the average systolic blood pressure value in the study group was significantly lower at posttest 3 (119.76±8.11) compared with the pretest (128.33±18.59; p<0.05). In contrast, the values were not significantly different in the control group: 125.95±20.48 (pretest) and 131.66±19.98 (posttest 3; p=0.068). The average respiratory rate in the study group was 22.07±3.85 during the pretest and 20.33±1.63 at posttest 3 (p<0.05), while in the control group, it was 27.69±6.96 at the pretest and 25.88±4.87 at posttest 3 (p=0.061).

**Table 2:** Hemodynamic monitoring in the experimental and control groups *p<0.05 (JRB1); **p<0.01; ***p<0.001

Variables	Study group, mean (SD)	F	P	Control group, mean (SD)	F	P
Systolic blood pressure						
Pretest	128.33 (18.59)	3.363	0.045*	125.95 (20.48)	3.508	0.068
Posttest 1	123.80 (16.52)			131.66 (19.98)		
Posttest 2	121.66 (13.77)			125.95 (20.48)		
Posttest 3	119.76 (8.11)			131.66 (19.98)		
Diastolic blood pressure						
Pretest	77.61 (10.54)	7.078	0.003**	80.00 (13.25)	11.795	0.001***
Posttest 1	77.14 (9.69)			85.00 (14.01)		
Posttest 2	80.00 (13.25)			80.00 (13.25)		
Posttest 3	85.00 (14.01)			80.00 (14.01)		
Heart rate						
Pretest	84.95 (15.08)	20.598	0.0001***	93.66 (21.10)	0.042	0.839
Posttest 1	83.71 (13.66)			93.38 (18.29)		
Posttest 2	93.66 (21.10)			93.66 (21.10)		
Posttest 3	99.04 (16.38)			93.38 (18.29)		
Respiration						
Pretest	22.07 (3.85)	4.451	0.019*	27.69 (6.96)	3.723	0.061
Posttest 1	20.85 (2.25)			25.88 (4.87)		
Posttest 2	21.57 (2.66)			27.69 (6.96)		
Posttest 3	20.33 (1.63)			25.88 (4.87)		

Subcomponents of sleep quality

Patients' perceptions of sleep quality in the ICU are presented in Table 2. The use of earplugs and eye masks in the study group significantly improved sleep depth (mean 67.86, standard deviation (SD)=14.90), sleep latency (68.81, SD=13.29), and quality of sleep (64.76, SD=15.96) on day 2 (posttest 2) and day 3 (posttest 3) compared with the control group.

The overall quality of sleep

A comparison of the overall sleep quality between the two groups revealed that the pretest mean for the study group was 43.73 (8.27) and the post-test on day 3 was 60.35 (6.85). In contrast, for the control group, it was 40.64 (8.67) on day 1 to 45.63 (6.95) on day 3, and it was significant at p<0.0001. The overall sleep quality in the study group (60.35±6.85; p=0.0001) was significantly better than in the control group (45.63, SD=6.95; p=0.003), as shown in Table 3.

**Table 3:** Subcomponents of Richards-Campbell sleep questionnaire scores before and after the intervention in the study and control groups **p<0.01; ***p<0.001

Group	Variables	Pretest, mean (SD)	Posttest 1, mean (SD)	Posttest 2, mean (SD)	Posttest 3, mean (SD)	Paired t-test (P)
Study group	Sleep depth	38.33 (17.52)	61.43 (19.45)	57.86 (17.07)	67.86 (14.90)	11.434 (0.0001***)
	Sleep latency	39.33 (19.13)	57.86 (13.17)	56.43 (14.45)	68.81 (13.29)	
	Awakening	35.24 (18.24)	60.24 (18.80)	66.90 (13.88)	63.57 (17.22)	
	Returning to sleep	42.86 (20.52)	57.62 (15.90)	65.00 (17.98)	61.67 (14.64)	
	Sleep quality	55.71 (17.41)	60.24 (14.23)	61.90 (15.81)	64.76 (15.96)	
	Noise	50.95 (19.61)	61.67 (14.80)	66.19 (15.92)	60.71 (17.72)	
Control group	Sleep depth	38.33 (17.52)	40.71 (20.17)	40.71 (12.95)	42.38 (15.27)	-3.096 (0.004**)
	Sleep latency	39.33 (19.13)	39.10 (17.17)	42.43 (20.05)	42.86 (14.53)	
	Awakening	35.24 (18.24)	40.95 (22.83)	42.38 (16.79)	46.43 (17.08)	
	Returning to sleep	42.86 (20.52)	39.76 (16.75)	40.24 (16.75)	45.48 (15.49)	
	Sleep quality	43.57 (19.98)	44.76 (19.41)	39.52 (18.34)	45.71 (17.69)	
	Noise	44.52 (20.74)	44.52 (20.74)	41.74 (18.57)	50.95 (21.95)	

Correlation between quality of sleep and fatigue

Concerning the perception of sleep with fatigue, it was found to be 0.0607 for the study group and 0.0711 for the control group at posttest 3 which was found to be negligible due to the shorter period of intervention and other confounding factors (Table 4).

**Table 4:** Overall perception of sleep quality and fatigue score between the study and control groups The F- and p-values were obtained from repeated measures analysis of variance. **p<0.01; ***p<0.001

Sleep and fatigue	Study group, mean (SD)	F	P	Control group, mean (SD)	F	P
Sleep quality						
Pretest	43.73 (8.27)	96.633	0.0001***	40.64 (8.67)	5.266	0.003**
Posttest 1	59.84 (7.50)			41.63 (8.26)		
Posttest 2	66.58 (6.56)			41.17 (7.11)		
Posttest 3	60.35 (6.85)			45.63 (6.95)		
Fatigue score						
Pretest	4.19 (1.64)	37.515	0.0001***	3.57 (1.93)	0.328	0.570
Posttest 1	3.40 (1.39)			3.42 (1.90)		
Posttest 2	2.09 (1.03)			3.57 (1.93)		
Posttest 3	2.21 (1.00)			3.42 (1.90)		

## Discussion

In the present study, 84 patients were assigned to a study group (n=42) and a control group (n=42) after 24 hours of admission to the ICU. All the patients completed posttest 3 on the third day of ICU stay. Patients in the study group received earplugs and eye masks as an intervention to reduce sleep disturbance, with an assessment of the impact on sleep quality and hemodynamic parameters.

The subcomponents of sleep quality, as measured with the Richards-Campbell sleep questionnaire, showed significant improvements for sleep depth, latency, awakening, returning to sleep, sleep quality, and noise for the study group at p<0.0001 and for the control group at p<0.004. The findings from this study are supported by a similar study by Arttawejkul et al. [19] that demonstrated a decrease in the arousal index and improved activity index among patients who received earplugs and eye masks for the first five nights in the ICU, compared with a control group. A clinical trial among 135 critical care unit (CCU) patients for three days after admission revealed that the institution of a quiet environment protocol and the use of simple, noninvasive, and cost-effective measures of earplugs and eye masks were beneficial for decreasing sleep disturbance and improving sleep efficacy, thereby improving sleep quality compared with the control group [20].

The present study showed significant improvements in sleep latency, sleep depth, and sleep quality for the intervention group. These results were similar to the findings of a cross-over study that compared the effects of exposure to simulated ICU noise and light, the use of earplugs and eye masks to the ICU noise and light, and the use of placebo and melatonin on sleep quality. That study found that the use of earplugs and eye masks was associated with improved perceived sleep quality, shorter sleep onset latency, reduced arousals and awakenings, and less anxiety [21]. In addition, a controlled clinical trial among 60 patients with acute coronary syndrome showed a significant improvement in total sleep quality with the use of an eye mask from the second night of admission till the time of discharge, compared with a control group [22]. The use of noise and light reduction strategies such as earplugs and eye masks led to better overall sleep perception and reduced prolonged awakenings among postoperative surgical ICU patients who had undergone breast-free flap surgery [23].

The present study showed a significant reduction in the hemodynamic parameters such as systolic blood pressure and respiration from the pretest to the third posttest, along with improvements in overall sleep quality for the study group compared with the control group. 

The present study showed that the intervention had an effect on fatigue, which was expected since sleep quality can influence the experience of fatigue. This significant reduction in fatigue was similar to findings reported by Ünsal and Demir [14], who showed that fatigue and sleep quality were interrelated among hospitalized surgical and internal clinic patients. In addition, a cross-over study to determine the effect of earplugs, eye masks, and ocean sounds identified that earplugs and eye masks were better than ocean sounds in improving sleep quality among ICU patients [24].

Several measures to improve sleep in the ICU have been evaluated, including the implementation of a protocol for scheduling specific nursing care activities and feeding before 10:00 pm and after 5:00 am, reducing chances of infusion alarms, lowering the alarm volume of monitors, and reducing the volume of staff communication. Such measures have been found to reduce sleep interruptions, support better overall sleep quality, and reduce daytime sleepiness [25]. A systematic review of nonpharmacological interventions in the CCU or ICU to reduce the effect of noise and light, such as the use of earplugs and eye masks, showed that these measures were associated with a lower incidence of delirium and significant improvements in total sleep time [26]. The findings of the present study are also supported by an observational study among 20 patients admitted to an interdisciplinary ICU, where the use of earplugs and eye masks was identified as being comfortable and facilitating sleep quality [27]. Noninvasive measures such as sound-absorbing and sound-masking techniques in combination with light reduction methods such as the use of eye masks have been found to enhance sleep in premature infants and ICU patients [28]. Simple nursing interventions that enhance comfort, rest, and sleep will enable patient recovery.

The current study had some limitations. Patients' self-reports on the Richards-Campbell sleep questionnaire and numerical fatigue scale were used. Three days of intervention were carried out as patients were transferred out of the ICU once they required less frequent monitoring. To overcome the limitations, three subsequent observations after the institution of earplugs and eye masks were carried out along with fatigue as another outcome measure on the three consecutive days that the patients were in the ICU. None of the experimental group patients reported any difficulty with the intervention.

## Conclusions

Continuous monitoring of patients is required in the ICU, yet sleep, rest, and comfort are crucial for healing to take place. Several measures have been identified as having an impact on the quality of sleep in the ICU, such as improving the infrastructural components, creating protocols and schedules for patient care activities, and increasing staff knowledge on noise and light control in the ICU. However, further reducing alarms and environmental noise may not be possible. The present study identified that the nonpharmacological cost-effective measure of earplugs and eye masks is beneficial in improving sleep and reducing fatigue among patients in ICUs.
